# Assessing the genomic evidence for conserved transcribed pseudogenes under selection

**DOI:** 10.1186/1471-2164-10-435

**Published:** 2009-09-15

**Authors:** Amit N Khachane, Paul M Harrison

**Affiliations:** 1 Department of Biology, McGill University, Stewart Biology Building, 1205 Docteur Penfield Ave., Montreal, QC, H3A 1B1 Canada

## Abstract

**Background:**

*Transcribed pseudogenes *are copies of protein-coding genes that have accumulated indicators of coding-sequence decay (such as frameshifts and premature stop codons), but nonetheless remain transcribed. Recent experimental evidence indicates that transcribed pseudogenes may regulate the expression of homologous genes, through antisense interference, or generation of small interfering RNAs (siRNAs). Here, we assessed the genomic evidence for such transcribed pseudogenes of potential functional importance, in the human genome. The most obvious indicators of such functional importance are significant evidence of conservation and selection pressure.

**Results:**

A variety of pseudogene annotations from multiple sources were pooled and filtered to obtain a subset of sequences that have significant mid-sequence disablements (frameshifts and premature stop codons), and that have clear evidence of full-length mRNA transcription. We found 1750 such transcribed pseudogene annotations (TPAs) in the human genome (corresponding to ~11.5% of human pseudogene annotations). We checked for syntenic conservation of TPAs in other mammals (rhesus monkey, mouse, rat, dog and cow). About half of the human TPAs are conserved in rhesus monkey, but strikingly, very few in mouse (~3%). The TPAs conserved in rhesus monkey show evidence of selection pressure (relative to surrounding intergenic DNA) on: *(i) *their GC content, and *(ii) *their rate of nucleotide substitution. This is in spite of distributions of Ka/Ks (ratios of non-synonymous to synonymous substitution rates), congruent with a lack of protein-coding ability. Furthermore, we have identified 68 human TPAs that are syntenically conserved in at least two other mammals. Interestingly, we observe three TPA sequences conserved in dog that have intermediate character (*i.e.*, evidence of both protein-coding ability and pseudogenicity), and discuss the implications of this.

**Conclusion:**

Through evolutionary analysis, we have identified candidate sequences for functional human transcribed pseudogenes, and have pinpointed 68 strong candidates for further investigation as potentially functional transcribed pseudogenes across multiple mammal species.

## Background

Pseudogenes (derived from protein-coding genes) are gene copies that show signs diagnostic of protein-coding deficiency. Such signs commonly include premature stop codons and coding-sequence frameshifts, or neutral codon substitution patterns [1,2]. Pseudogenes can arise in two chief ways: *(i) *from retrotransposition, *i.e.*, reverse transcription of a cellular messenger RNA, followed by reintegration into the genomic DNA [3-5], or *(ii) *from decay of genes that originated (however long ago) from duplication [1,6]. These genomic entities have generally been believed to be non-functional. Historically, there were some early individual reports of transcribed pseudogenes in the scientific literature [2,7,8]. Examples included: human leukocyte interferon (LeIFN) pseudogene [9], glyceraldehyde-3-phosphate dehydrogenase pseudogene [10], glucocerebrosidase pseudogene [11], steroid 21-hydrolase pseudogene [7], glutamine synthetase pseudogene [12], tumor repressor ΨPTEN [13].

More recently, genome-wide screens have detected transcription evidence for many retropseudogenes (>200) in humans [14-17]. In mouse oocytes, transcribed pseudogenes have been shown to play a significant role in the generation of small interfering RNAs (siRNAs) [18,19], which regulate the expression of homologous genes.

Collectively, these reports indicate that an unknown cohort of human transcribed pseudogenes could be potentially functional in regulation of gene transcription. A key indicator of such function is significant conservation in other mammalian genomes. Svensson *et al*. [20] have explored conservation of apparent pseudogenes in three mammals (human, chimpanzee and mouse). Their analysis revealed 30 cases of transcribed pseudogenes that are preserved in mouse. Here, we analyze the distribution of transcribed pseudogene annotations (TPAs) in the human and mouse genomes, examine their conservation in an expanded panel of mammals (rhesus monkey, mouse, rat, dog and cow), and assess evidence for significant selection pressures. TPAs that are conserved in rhesus monkey show evidence of significant selection pressure, despite also displaying codon substitution patterns characteristic of non-protein-coding sequences. Also, we have discovered a short-list of 68 putative human transcribed pseudogenes that are syntenically conserved in at least two other mammals from our panel. These sequences represent a strong subset of candidates for further investigation as functional transcribed pseudogenes.

## Results & Discussion

### Derivation of transcribed pseudogene annotations (TPAs) in the human genome

To identify transcribed pseudogenes, transcript sequences from the Unigene, RefSeq and H-InvDB databases were mapped onto the human genome and were examined for overlap with pseudogene annotations. These pseudogene annotations were taken from the VEGA  and  websites (see *Methods *for details). We pooled these datasets with re-mappings of: *(i) *'disrupted mRNAs' (dmRNAs) [16], and *(ii) *transcribed processed pseudogenes [14], from previous analyses [14,16]. Overlap of the transcripts with pseudogenes was verified through using the positions of mid-sequence disablements (frameshifts and premature stop codons) as 'anchors'. That is, at least one disablement position was required to occur in both the genomic DNA and the transcript sequence (see *Methods *for further details).

We found that ~11.5% (1750/15000) of human pseudogenes are transcribed (after correcting for pseudogene annotation overlaps within and between the various data sets) [see Additional file 1]. Table 1 summarises the numbers of transcribed pseudogene annotations (TPAs) in different categories and data sets. The number of processed pseudogenes that were identified to be transcribed is 3-4 times higher than in our previous analysis [14]. Interestingly, in humans, regardless of category, only a small fraction of TPAs are transcribed completely in the antisense direction (~3-6%). Such a finding of significant avoidance of antisense transcription (Table 1) is surprising, especially for retropseudogenes. Retrotransposed sequences are inserted back into the genomic DNA irrespective of the position of existing local promoters. Thus, one would expect equal numbers of sense and antisense transcripts. However, the above finding indicates a general selection pressure against antisense transcribed pseudogenes, thus generally limiting the possibilities for complementary hybridization with transcripts and RNA elements from homologous genes.

**Table 1:** Percentages of TPAs in human and mouse.

**Dataset**	**Transcribed (human)**						**Transcribed (mouse)**	
**VEGA**	Total = 866/8160 (10.6%)		# Processed = 383/3737 (10.24%)		# Non-processed = 71/1078 (6.58%)		Total = 71/4187 (1.7%)	
	
	828 (sense)	38 (antisense)	371 (sense)	12 (antisense)	61 (sense)	4 (antisense)	49 (sense)	22 (antisense)

****(excluding ambiguous pseudogenes)	Total = 1035/13354 (7.75%)		# Processed = 767/11072 (6.93%)		# Non-processed = 268/2282 (11.74%)		Total = 65/15064 (0.5%)	
	
	977 (sense)	58 (antisense)	724 (sense)	43 (antisense)	253 (sense)	15 (antisense)	53 (sense)	12 (antisense)

We performed a similar survey in the mouse genome for TPAs. Surprisingly, in the mouse genome, we found a very low percentage of TPAs, in comparison to the human genome (<2%) (P << 0.001 for the likelihood of the number in mouse, given the human percentage as an expectation, using binomial statistics). This is despite these two animals having similar amounts of pseudogene annotation data (Table 1), and transcript data (203,785 transcript sequences in total for human, and 203,550 for mouse). This indicates that transcribed pseudogenes are rarer in mice than in humans.

### Identification of orthologous pseudogenes in mammals

Transcription of pseudogenes *per se *does not necessarily indicate functionality. It has been shown that transcriptional activation at a particular genomic locus has a ripple effect on the neighboring loci [21]. It is therefore possible that many transcribed pseudogenes arise simply because of this. However, of the various identified human TPAs in our present study, those that are evolutionarily conserved across mammals due to natural selection are more likely to be biologically functional. Therefore, we set out to identify a list of such orthologous transcribed pseudogenes that have conserved in ≥2 of our panel of mammals.

Certain gene families are known to spawn large numbers of pseudogenes. Examples include olfactory receptors [22], ribosomal-protein genes [23], human thioredoxin and glutaredoxin [24], ABC transporters [25], and heat shock proteins [26]. In such cases, identifying orthologs in other mammals using the standard bi-directional best-hit procedure is problematic, since the rates of sequence evolution may vary in different lineages and genomic regions. Furthermore, such a procedure does not work well for pseudogenes, since these sequences are not evolving like protein-coding genes, which are under strong purifying selection. Because of this, the best match obtained using *blastp *to a pseudogene query is expectedly the parent protein-coding gene or a pseudogene recently evolved from the parent gene. Thus, the standard bi-directional best-hit procedure alone is not sufficient. Therefore, here, we have used synteny information between two organisms to pin-point pseudogene orthologs. We have used synteny maps along with homology-based searches to identify conserved orthologs in five mammals (rhesus monkey, mouse, rat, dog and cow) (see *Methods *for details). We identified a set of 68 human TPAs that are conserved in at least two of these mammals, representing potentially functional candidates (see Additional file 2: Table S1). In general, although approximately half (742/1750) of the human TPAs are syntenically conserved in rhesus monkey, only 3% are syntenically conserved in mouse. This suggests that a large number of human transcribed pseudogenes are primate-specific.

**Figure 1:**
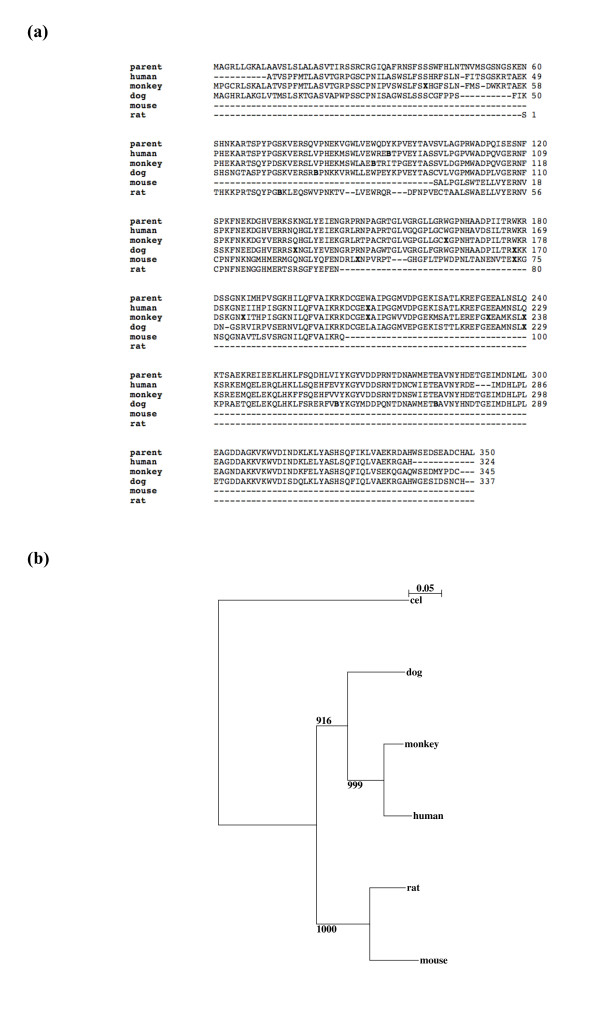
**Multiple sequence alignment and phylogenetic analysis of a human transcribed pseudogene that has orthologs in the ≥2 other mammals**. (a) Multiple sequence alignment of conceptually-translated ortholog sequences (*urn:lsid:pseudogene.org:9606.Pseudogene:4346*; see Additional file 2: Table S2) from different mammals along with the human parental protein sequence (human ADP-ribose pyrophosphatase, swissprot accession id: NUDT9_HUMAN). The positions of stop codons in the alignment are denoted by '**X**' and frameshifts denoted as '**B**'. (b) A rooted phylogenetic tree constructed from the most conserved segment from a multiple nucleotide sequence alignment between ortholog sequences (human parental protein sequence - ADP-ribose pyrophosphatase, swissprot accession id: NUDT9_HUMAN). As an outgroup, we chose a protein sequence from *C.elegans *from the 'nudix' hydrolase family but belonging to another subfamily (NDX2_CAEEL) identified based on BLAST matching. PHYLIP Bootstrap support values out of 1000 iterations are indicated at each node.

**Figure 2:**
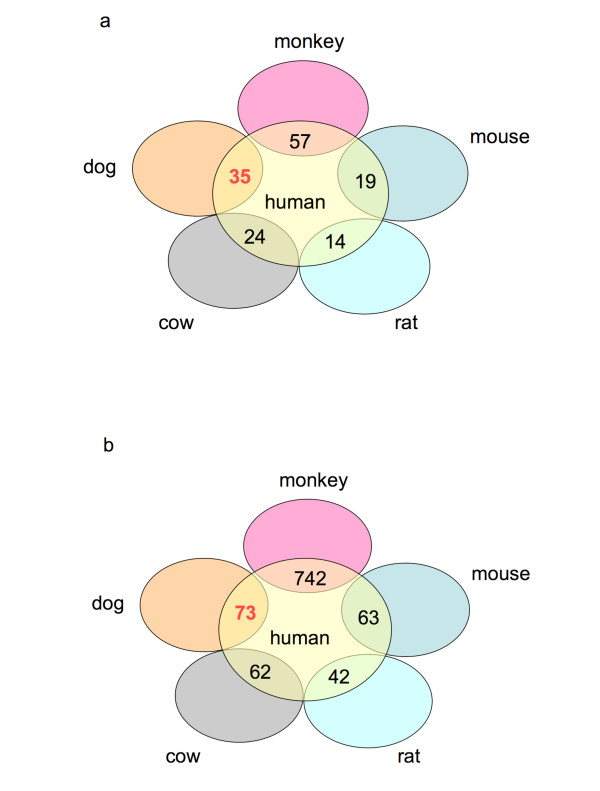
**Number of shared orthologs between human and other mammals**. (a) For the 68 conserved TPAs (orthologs in > = 2 mammals); (b) for all conserved TPAs between human and the other mammals. The shared number of cases between dog and human is highlighted in red to indicate that this number is higher than for human/rodents.

A multiple sequence alignment of orthologous sequences for an example taken from Additional file 2: Table S2, is shown in Figure 1(a). The corresponding phylogenetic tree is drawn in Figure 1(b). This example is a human pseudogene named '*urn:lsid:pseudogene.org:9606.Pseudogene:4346*', that is homologous to human ADP-ribose pyrophosphatase. In this case, one can see clearly that disablements at several positions in the alignment are conserved in divergent species, and parsimoniously would be assigned in the ancestral sequence. Also, in this phylogenetic tree, dog clusters closer to primates, than rodents do; this may be due to variance in local genomic mutation rates. Interestingly, we find that a significantly higher number of human transcribed pseudogenes were conserved in dog, compared to in mouse (Fisher's exact test, *P*-value: 0.0086) (Figure 2). There is some debate regarding whether human is phylogenetically closer to rodents than to dog, although most data analysis indicates a rodent-primate grouping [27-29].

### Conserved transcribed pseudogenes are overrepresented on chromosome X

**Figure 3:**
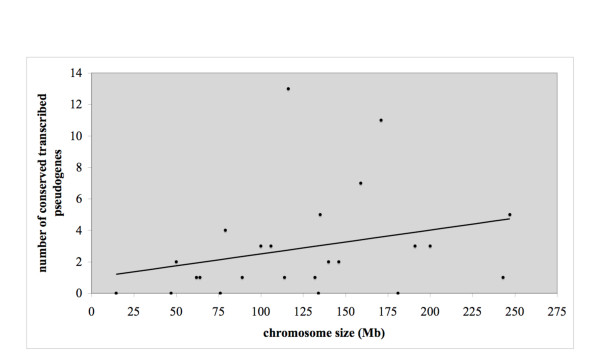
**A scatter plot showing the number of conserved TPAs on each human chromosome versus the chromosome size (in Mb)**. The chromosome X is circled. The collective size of the human genome, excluding chromosome X, is 3,098,124,053; and the size of the chromosome X after normalizing for chromosome dosage in gametes, as done by Emerson *et al*. [30], is 116,185,316 (*i.e*., 0.75 times the original size of 154,913,754), which harbors 13 transcribed preserved pseudogenes. Since the collective genome size is 27 times bigger than that of the human chromosome X, we expect 2 or 3 preserved pseudogenes on the chromosome X. Presence of 13 cases, an increase of more than 4.5 times than expected, suggests a statistically significant overrepresentation (exact binomial test of goodness-of-fit, *P*-value < 10^-5^, for the null hypothesis (*H*_0_) that there is no difference between the observed and expected frequencies of transcribed preserved pseudogenes on the chromosome X).

It is noteworthy that the highest number of conserved TPAs is on the human chromosome X (13 out of the total of 68; Figure 3 and Additional file 2: Table S2), followed by 11 cases on chromosome 6. There is also a significant over-representation of human conserved TPAs on these chromosomes after normalizing for the chromosome size and dosage in gametes (χ^2 ^test, d.f. = 1, *P*-value < 10^-3^). Furthermore, it is chromosome X that is consistently and most significantly overrepresented in the whole population of TPAs, and in the datasets of TPAs that are conserved in monkey and in mouse (calculations not shown). This finding is in line with the observation that over the course of evolution there has been some extensive gene trafficking to/from the mammalian X chromosome [30].

### Analysis of TPAs for selection to maintain them

**Figure 4:**
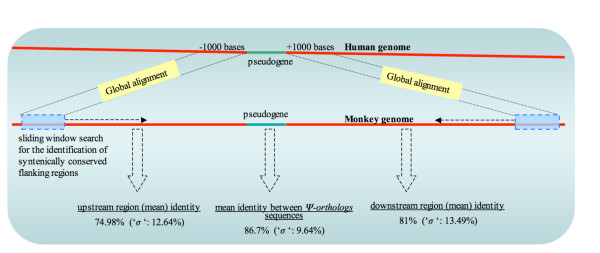
**A schematic representation of the procedure for identifying syntenic noncoding (flanking) regions**. Flanking regions (10000 nts) are scanned in a sliding window, equal to the length of conceptual human pseudogene transcript), by globally aligning it to the upstream/downstream regions of the human pseudogene. The best scoring window is identified, which corresponds to the syntenic (flanking) region in the monkey genome.

Human TPAs from the VEGA data set, that have syntenically-conserved orthologs in rhesus monkey, were analyzed for significant selection pressure to maintain them. This was assessed through comparison of the nucleotide percentage sequence identity between orthologs, with the highest nucleotide percentage sequence identity for the immediately flanking genomic regions, as illustrated in Figure 4. We chose the VEGA data set for this analysis, since the genomic coordinates of this pseudogene annotation data set are more precisely annotated.

Our analysis indicated that TPA orthologs have higher sequence homology in comparison to their syntenic flanking regions. Average sequence identities among different syntenic regions in human and rhesus monkey are as follows: 75.0% (s.d. 12.6%) in the 5' areas, 81.0% in the 3' areas (s.d. 13.5%), and 86.7% (s.d. 9.6%) in the TPA sequences. The difference in the sequence identities between pseudogenes and the respective flanking regions is statistically significant (Wilcoxon signed rank test, *P*-value: < 5e^-50^). In the majority of cases (~86%, 293/341), the percentage sequence identity between orthologous TPA sequences is greater than that of the flanking regions. This suggests that significant selection pressure exists to maintain them. We note that similar analysis comparing the human and chimpanzee genomes is not informative because the species are too similar. Also, comparisons of the human genome with the other mammals in our panel are not informative either, because the appropriate regions cannot be aligned accurately or significantly.

### Conserved TPAs tend to be GC rich

We examined whether there existed any sequence feature that distinguished conserved TPAs from the rest of the human pseudogene population. A positive finding would indicate that these pseudogenes are not evolving neutrally. It has been widely observed that genes tend to be GC-rich in comparison to non-transcribed genomic segments [31]. Here, we examined whether TPAs and annotations of non-transcribed pseudogenes showed any difference in the GC contents relative to their flanking regions (GC_diff_). Pseudogene populations from a variety of organisms have been shown to relax to the composition of intergenic DNA over evolutionary time [32,33]. Here, the GC content of neutrally-evolving pseudogenes would be expected to relax to that of the background genomic GC content. Interestingly, GC content calculations revealed that 84% (327/391) of the human TPAs derived from the VEGA pseudogene data set, that are conserved in rhesus monkey, have GC content greater than their flanking regions (Table 2). This compares to 74% (5289/7154) for the non-transcribed cases (Table 2). This difference is statistically significant (χ^2 ^test, d.f. = 1, *P*-value < 10^-4^). A similar result is obtained if we examine the whole population of TPAs, or also if we just examine transcription of conserved *processed *pseudogenes. There is however no such significant differences for transcribed pseudogenes of the 'nonprocessed' and 'unclassified' pseudogene categories (Table 2). This shows that there is a greater tendency for the conserved TPAs to be GC-rich than for non-transcribed cases, and that this tendency arises primarily because of transcription of processed pseudogenes. This finding on GC content is further evidence that such transcribed pseudogenes are not evolving neutrally. Such GC trends can be explained by selection for transcriptional efficiency, as noted above [31].

**Table 2:** Proportions of pseudogenes that have GC contents higher relative to their flanking regions in different categories of VEGA annotated human pseudogenes.

**Pseudogene category**	**Transcribed and conserved (monkey) pseudogenes**	**Non-transcribed pseudogenes**	*P*-value for statistical difference*
**Processed**	165/188 (87.77%)	2473/3274 (75.53%)	< 10^-04^

**Nonprocessed**	9/14 (64.29%)	577/910 (63.40%)	0.7807

**Unclassified**	153/189 (80.95%)	2239/2970 (75.39%)	0.0961

**Total**	327/391 (83.63%)	5289/7154 (73.93%)	< 0.0001

* χ^2 ^test with *d.f*. = 1.

We checked whether the age of pseudogenes could be causing the GC content differences noticed above. To do this, we looked at the GC_diff _in the following (VEGA) subsets of TPAs, *i.e.: (i) *TPAs unique to humans; *(ii) *TPAs conserved in rhesus monkey only; *(iii) *TPAs conserved in more divergent mammals such as mouse, rat, dog and cow. We found that 82.5% (381/462) of set (i) have GC_diff _> 0 (*i.e.*, GC content of pseudogene greater than that of the flanking region). Similar percentages were observed in the other classes: 84.1% (317/377) in set (ii), and 77.78% (21/27) in set (iii). There was no statistically significant difference in the GC_diff _between any two of the classes (χ^2 ^test, *P-value *> 0.55), suggesting that age of pseudogenes does not have any influence on the observed GC content differences.

### Ka/Ks trends for TPAs that are conserved in rhesus monkey

We decided to assess the genomic evidence for a lack of protein-coding ability in the human TPAs that are syntenically conserved in rhesus monkey. We compared TPA characteristics to the characteristics of two other groupings: *(i) *known human protein-coding genes with orthologs in rhesus monkey; *(ii) *populations of simulated sequences that are randomly mutating without coding-sequence selection pressures. The human TPA sequences are used as starting sequences for these simulations. The protocol for these simulations is described in '*Methods: Ka/Ks ratio calculations*'.

**Figure 5:**
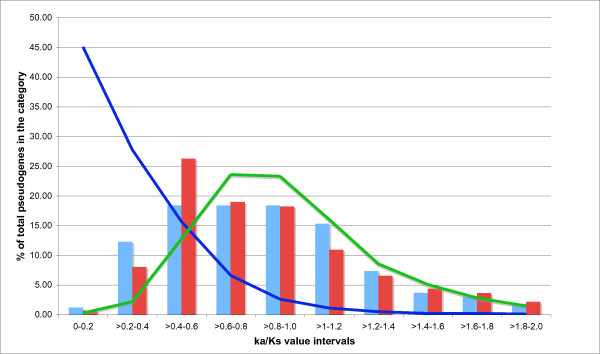
**Distributions of Ka/Ks for TPA_DD_s (blue bar), and TPA_NDD_s (red bar)**. Also shown are: the distribution of Ka/Ks for simulated sequences that have randomly mutated without coding-sequence selection pressures (green curve; derived as described in *Methods*), and the Ka/Ks distribution for orthologous pairs of known protein-coding genes from rhesus monkey and human.

The ratio of non-synonymous to synonymous substitution rates (Ka/Ks) provides a measure of selection pressure for protein-coding ability on nucleotide sequences. Classically, values significantly <<1.0 indicate purifying selection, whereas sequences without coding ability theoretically yield values near 1.0. We examined the trends for Ka/Ks in the population of human TPAs that are conserved syntenically in the rhesus monkey. Ka/Ks was calculated using the PAML package (as described in *Methods*). The distribution of Ka/Ks is shown for TPAs, split into two groups, those that have a disrupted protein domain of known three-dimensional structure (TPA_DD_, blue bar, Figure 5), and those that do not (TPA_NDD_, red bar, Figure 5).

In addition, we calculated the distribution of Ka/Ks values for sequences that are randomly mutating without coding-sequence selection pressures. These sequences were generated using the simulation protocol described in '*Methods: Ka/Ks ratio calculations'*, using the human TPAs as starting sequences (green curve, Figure 5). The Ka/Ks distribution for these simulated sequences does not peak at ~1.0, as would be classically expected. This is likely due to some inaccuracy in modeling the expected frequency for the different possible nucleotide substitutions, which varies for different genomic areas [3]. The distribution for TPA_DD_s peaks in the range 0.6 to 1.0. This peak is similar for the randomly-mutating sequences (Figure 5). For the TPA_NDD_s, the peak is at lower Ka/Ks values (0.4-0.6).

As a further comparison, we have calculated the Ka/Ks curve for orthologous pairs of protein-coding genes from the rhesus monkey and the human (blue curve, Figure 5). Clearly, these protein-coding sequences behave very differently from the TPAs, with a substantial mode in the range 0.0 to 0.2. In summary, these Ka/Ks trends indicate that the substitution patterns in the TPAs generally behave like non-protein-coding sequences, and *not *like protein-coding ones. This is despite the overall significant conservation relative to surrounding intergenic genomic DNA that was discussed in the previous section.

### Analysis of the ratio of non-synonymous to synonymous substitution rates (Ka/Ks) relative to orthologous TPAs in dog and in mouse

**Figure 6:**
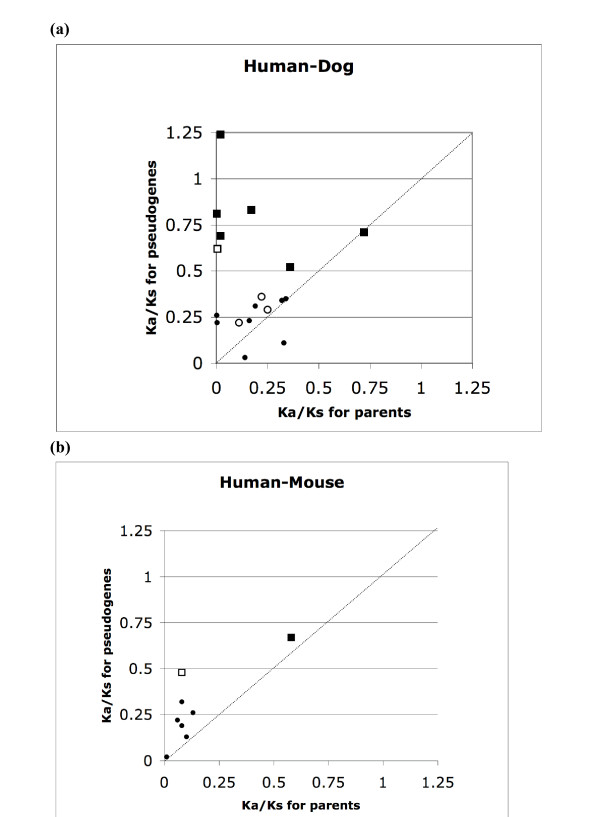
**Scatter plots showing Ka/Ks ratio comparisons between TPA sequences and their respective orthologous parental protein coding genes for: (a) human/dog comparisons, (b) human/mouse comparisons**. Ka/Ks values for TPAs, that are significantly less than values for neutral selection determined by simulation, are indicated as circle symbols, else the Ka/Ks values are indicated with square symbols. Those TPAs that have a disrupted protein domain of known three-dimensional structure are indicated with unfilled symbols, those without such a domain are indicated with filled symbols.

To gain a more complete picture, we also examined Ka/Ks values for TPAs that are conserved in two more divergent species, the dog and the mouse. We compared Ka/Ks values for orthologous TPA pairs (termed Ka/Ks_*Ψ*-*ortho*_), with the corresponding Ka/Ks values for their parent genes (Ka/Ks_*parent*-*ortho*_) (Figure 6). These were calculated for human/dog (Figure 6(a)), and human/mouse comparisons (Figure 6(b)). For human/dog comparisons, the substantial majority (83%) have Ka/Ks_*Ψ*-*ortho *_> Ka/Ks_*parent*-*ortho*_, whereas for human/mouse all of the pseudogene pairs have larger Ka/Ks values than their corresponding parent pairs.

The Ka/Ks results suggest that these transcribed pseudogenes are relaxing to higher Ka/Ks values, since origination from their parents. But why do they not have Ka/Ks values of ~1.0? We suggest that this is chiefly because: *(i) *there may be some inaccuracy in modelling the expected frequency for the different possible nucleotide substitutions, which varies for different genomic areas (as noted in the previous section); *(ii) *in some cases, the transcribed pseudogenes were originally protein-coding, and became disabled subsequently in multiple lineages; *(iii) *some of them maintain an imprint of the original coding sequence because of selection pressure for regulation of homologous genes *via *antisense interference (*e.g.*, through genesis of siRNAs); *(iv) *selection pressures on non-synonymous codon substitution rates in protein-coding genes, may have relaxed in the pseudogenes, contributing to an apparent relative increase in Ks; *(v) *it is also possible that some of these sequences are currently protein-coding, and have evolved through multiple coding-sequence disablements, as discussed previously [4].

To examine these data more closely, we calculated whether the Ka/Ks_*Ψ*-*ortho *_values are significantly less than would be expected for mutation without coding-sequence selection pressures (using the simulational analysis described in the *Methods *section). Several cases with such significant values (that may indicate purifying selection typical of protein-coding sequences), are observed (represented by circles in the Figure 6 plots). These Ka/Ks values (that apparently indicate protein-coding ability) may arise for the reasons listed in the preceding paragraph.

In addition, we examined whether the TPAs contain a protein domain of known three-dimensional structure, that is disabled by a frameshift or a premature stop codon (denoted 'TPA_*DD*_s'; see *Methods *for details of annotation of such domains). The TPA_*DD*_s are indicated by unfilled symbols in parts (a) and (b) of Figure 6. Interestingly, in the human-dog comparisons, there are three cases of TPA orthologous pairs that have such a disabled protein domain, despite Ka/Ks values that indicate apparent purifying selection. These sequences are thus of 'intermediate' character, *i.e.*, they have evidence of both protein-coding ability and pseudogenicity.

### Antisense homologies of human pseudogenes to other full-length human cDNAs

Transcribed pseudogenes can regulate the expression of other genes by RNA interference mechanisms. For example, antisense transcribed RNA from a NOS pseudogene regulates the expression of neural nitric oxide synthase (nNOS) protein through formation of RNA duplex [34,35]. Therefore, we investigated how many of the TPAs have antisense homology to the annotated full-length human cDNAs (*E*-value < 1e^-10 ^and alignment length > = 100 nucleotides). A small proportion (8.3%, 69/828) of the human (VEGA) pseudogenic transcripts have either complete or partial, but significant, reverse complement (antisense) homology to human cDNAs. Of these, 63 have short length strong antisense homology to human cDNAs (alignment length > = 20, mismatches < = 2). However, there is no significant association of such antisense homologies to pseudogene transcription, since non-transcribed pseudogenes have similar levels of antisense homology (7.65%, χ^2 ^test *P*-value = 0.5).

Out of the identified 68 human conserved TPAs, 3 have antisense homology to human cDNAs (*E*-value < 1e^-10^, 5 if alignment length > = 50 nucleotides is considered) (Table 3). These are cases that may generate small interfering RNAs (siRNAs) that could potentially regulate the expression levels of their homologous genes. Pseudogenes have been implicated in the negative regulation of parental genes (for a review, see ref. [2]) and in the *Dicer*-mediated generation of small RNAs [18,19]. It would be interesting to verify experimentally whether these pseudogene transcripts can indeed generate small interfering RNAs, through the action of *Dicer*.

**Table 3:** Human conserved TPAs that have antisense homology to human full length cDNAs.

**Pseudogene/transcript id**	**ENSEMBL transcript id**	**Antisense identity (%)**	**Alignment length**	***E*-value**
OTTHUMT00000269970	ENST00000323294	94.92	118	7.00e-46

OTTHUMT00000270027	ENST00000379565	87.59	282	2.00e-70

chr10_Q96RG0.4_-	ENST00000315032	83.23	161	1.00e-17

urn:lsid:pseudogene.org:9606.Pseudogene:18315	ENST00000344386	96.3	81	3.00e-31

OTTHUMT00000082689	ENST00000343936	91.38	58	2.00e-12

### Small RNA mappings to pseudogenes

Transcribed pseudogenes can also regulate the transcription of genes by producing siRNAs that have antisense homology [18,19]. Due to unavailability of genome-wide human siRNA data, we used the siRNA data for the mouse genome from Tam *et al*. [19] and Watanabe *et al*. [18] to check how many of the small RNAs mapped to mouse transcribed pseudogenes that we identified. Interestingly, 24 out of 136 (17.6%) mouse TPAs had siRNA mappings compared to ~1% (178/18168) of the total mouse pseudogenes. The above difference is statistically significant (*P*-value < 0.05, using normal statistics for the distribution of the mean number of transcribed pseudogenes in a sample of 136 cases). This demonstrates that transcribed pseudogenes are significantly likely to generate siRNAs in mouse. For comparison, in *Arabidopsis thaliana*, ~40% of 572 pseudogenes have small RNA mappings [36].

## Conclusion

In this study, we identified hundreds of cases of putative transcribed pseudogene annotations (TPAs), in the human genome. Importantly, we detected evidence for selection pressure on these transcribed elements. These findings therefore draw wider attention towards the potential functionality of these genomic elements. In addition, we found that 68 human TPAs are conserved in at least 2 other studied mammals. These human TPAs have ancient origins dating back >120 million years ago, as evidenced by their conservation patterns across distantly related mammals. These pseudogenes represent novel genomic elements of potential functional relevance.

We have shown that human TPAs that are syntenically conserved in rhesus monkey generally behave like non-protein-coding sequences, despite significant selection pressure on them, relative to the surrounding genomic DNA. Examination of Ka/Ks values for TPAs that are conserved in more divergent species (mouse and dog), indicated that some TPAs might actually be protein-coding. However, we cannot rule out other reasons for these low Ka/Ks values. For example, it is possible that some of these sequences had phases of protein-coding ability at some evolutionary stage. Also, it is possible that there is an imprint of purifying selection on these sequences because of selection pressure to form small interfering RNAs with homologous protein-coding genes. Ultimately, these questions can only be answered by detailed experimental characterization of these molecules; our analysis here provides a rich data source for prioritizing likely candidates of functional importance as transcribed pseudogenes.

## Methods

### 1. Collection of data

Complete genome sequences of mammals were obtained from  (Ensembl release 47 for human genome; Ensembl release 48 for other mammals, namely, rhesus monkey, mouse, rat, cow and dog). Pseudogene annotations for both processed and nonprocessed categories, were obtained from [; [37,38]] and for VEGA pseudogenes from , for disrupted mRNAs (dmRNAs) from Harrison and Yu [16] and for other transcribed processed pseudogenes from Harrison *et al*. [14]. The Blastx program [39] was used to determine the parent protein coding genes for VEGA pseudogenes (using *E*-value < 1e^-09 ^as significance threshold), whereas for other datasets the annotations were readily available at the respective websites mentioned above.

### 2. Screening for putative transcribed pseudogenes

**Figure 7:**
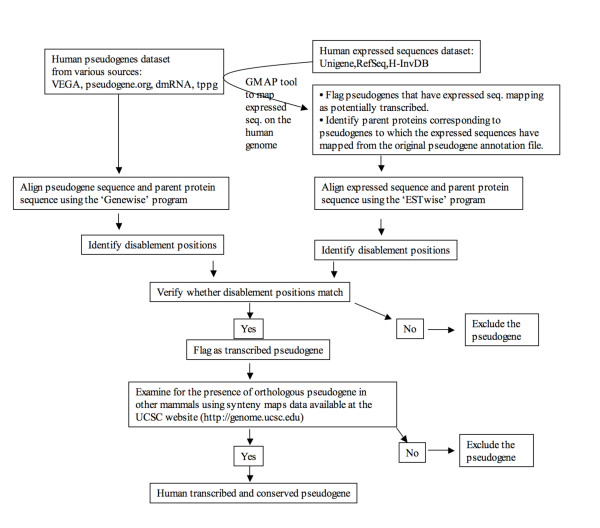
**Annotation pipeline for human transcribed and conserved pseudogenes**. (Note: 'dmRNA' represents disrupted mRNA dataset from ref. [16], 'tppg' represents transcribed processed pseudogenes from ref. [14].)

Transcription data for human and mouse were taken from RefSeq database [40], Unigene database at the NCBI , H-InvDB database  and Fantom3 database . To identify putative transcribed pseudogenes, individual transcript sequences were mapped onto the respective genomes using GMAP software [41] with match criteria of >99% sequence identity and >99% sequence coverage. Transcript sequences that mapped to pseudogenes were aligned to parent protein sequences of respective pseudogenes to identify disablements such as frame shift or premature stop codon using the 'GeneWise' program (Wise2 - version 2.1.20 package downloaded from the European Bioinformatics Institute, ) [42]. The disablement positions in pseudogenes and transcript sequences were then used as 'anchors' to confirm the transcription of pseudogenes as in previous analyses [14,15,43]. Additional data file (file 1) contains the list of transcribed human pseudogenes. For a schematic representation of the annotation pipeline, see Figure. 7.

### 3. Identification of orthologous pseudogenes in various sequenced mammalian genomes

Orthologous counterparts to a human pseudogene are detected by the presence of a homologous sequence at the syntenic position in the other mammalian genome. Based on this criterion, a search was carried out within 100 kb nucleotides distance of the exact syntenic coordinate (because genes can shuffle locally) in the target mammal as indicated in the synteny maps, to locate the orthologous pseudogenes. 'GeneWise' tool [42] was used, to align the above-obtained genomic DNA sequence and the human parental protein sequence, and to detect disablements in the alignment. The following mammals were included in the analysis: monkey, mouse, rat, cow and dog. The pair wise synteny map data for the various mammals were obtained from .

### 4. Analysis for pseudogene sequence conservation

Flanking sequences 5' and 3' of human pseudogenes were individually obtained, of length equal to the length of the human pseudogene, and were each globally aligned using 'needle' module of EMBOSS package  to the corresponding flanking region sequences (10000 nucleotides 5' and 3') of monkey in a sliding window of size also equal to the length of human pseudogene. The window in which best identity score was obtained was considered as the most optimum alignment between the flanking regions, representing syntenic regions. The Wilcoxon signed rank test was used for assessing the statistical significance of the difference between the degrees of homology calculated between two orthologous pseudogenes and that between the respective (orthologous) flanking regions. Cases with pair wise sequence identities <40% were excluded.

### 5. Analysis of lengths and GC percentage of pseudogenes and their flanking regions

For sequence length and GC percentage calculations, only the exonic segments of pseudogenes were considered. One thousand nucleotides upstream and downstream of a pseudogenes were considered as flanking regions. GC content is calculated as the sum of guanine and cytosine nucleotides divided by the total number of nucleotides represented in terms of percentage.

### 6. Ka/Ks ratio calculations

'PAL2NAL' [44] was used to construct codon alignments between protein sequences (conceptual amino acid translation sequences in the case of pseudogenes) and corresponding DNA sequences, separately, for orthologous pseudogenes and parental protein coding genes. 'PAML 4' package [45] was used to calculate Ka/Ks ratios. Orthologs of human parental protein coding genes were identified using a similar approach as that for pseudogene orthologs discussed above, and also obtained from Ensembl database.

We derived a simulation protocol to calculate Ka/Ks values for evolution without coding-sequence selection pressures. This simulation protocol is as follows: *(i) *the nucleotide distance (D_nt_) between a sequence and its ortholog was calculated, using the program DNADIST [46]; *(ii) *for each sequence, samples of 500 simulated sequences were generated, by randomly mutating the human sequence until the D_nt _value was reached; *(iii) *Ka/Ks was calculated using PAML [45], for each simulated sequence compared to the original human sequence; *(iv) *those original human sequences that have Ka/Ks values < 95% of simulated Ka/Ks values were labeled as potentially under significant purifying selection. For these simulations, all Ka/Ks calculations are performed on the longest ORF in the sequence.

We also analysed simulated distributions of Ka/Ks for populations of sequences mutating without coding-sequence selection pressures, starting from the human TPA sequences. These were derived simply by merging the simulated distributions of Ka/Ks for each individual TPA.

### 7. Annotation of disrupted protein domains

Protein domains were assigned to the TPAs, using protein structure domain sequences downloaded from the ASTRALSCOP database , as described previously [4]. Protein domains sequences were aligned to the TPA nucleotide sequences to assess for disablement by a frameshift or premature stop codon at least 15 amino acids from the end of the aligned subsequence. Disablements were required to be detected both by blast/bl2seq and by the TFASTX program [4,39].

### 8. Antisense homology

Transcribed human pseudogenes were aligned to full-length annotated human cDNA to examine for any antisense homology by using the sequence-searching program BlastN from the BLAST package (*E*-value < 1e^-10^).

### 9. small RNA (siRNA) mapping

siRNAs have been previously determined in the mouse genome [18,19]. Using this data we mapped the siRNA sequences onto the mouse genome using GMAP software [41], and checked how many of these overlap with the annotations of transcribed mouse pseudogenes.

### 10. Phylogenetic analysis

Ortholog sequences to the human transcribed ADP-ribose pyrophosphatase pseudogene (urn:lsid:pseudogene.org:9606.Pseudogene:4346; see Table 1), were obtained from the various studied mammals and were aligned using the online ClustalW tool . The most conserved segment representing 257-396 positions of the human pseudogene was considered for the phylogenetic analysis. Phylogenetic tree was constructed using 'PHYLIP' software [46]. The tree was evaluated statistically using 1000 bootstrap iterations and was visualized using the 'NJplot' tool [47].

## Supplementary Material

Additional file 1 **List of transcribed human pseudogenes**. Genomic coordinates of the transcribed pseudogenes found in the human genome. Click here for file

Additional file 2 **Table S2**. List of human TPAs that are conserved in other mammals. Click here for file
