# Supplementary material for: Assessing the genomic evidence for conserved transcribed pseudogenes under selection
Source: BMC Genomics. 2009 Sep 15;10:435. doi: 10.1186/1471-2164-10-435 (PMC2753554; doi:10.1186/1471-2164-10-435)
Supplement: Additional file 2 — Table S2. List of human TPAs that are conserved in other mammals. [file 1471-2164-10-435-S2.doc]

**Table 2:** List of human TPAs that are conserved in other mammals.

| **Pseudogene/transcript identifier** | **Parent protein accession** | **Chromosome number** | **Start coordinate on chromosome** | **End coordinate on chromosome** | **Strand** | **Conservation** |
| --- | --- | --- | --- | --- | --- | --- |
| OTTHUMT00000040170 | ZSC12_HUMAN | 6 | 28168305 | 28168598 | 1 | MDC |
| OTTHUMT00000074738 | ADA21_HUMAN | 14 | 69781298 | 69784837 | -1 | MDR |
| OTTHUMT00000285230 | Q8N9J8_HUMAN | 17 | 11405799 | 11408106 | 1 | MDC |
| OTTHUMT00000026862 | SERC_HUMAN | 1 | 79293218 | 79294328 | -1 | MR |
| OTTHUMT00000033089 | A8MYG3_HUMAN | 1 | 116201108 | 116201963 | -1 | MD |
| OTTHUMT00000039805 | RL7A_HUMAN | 6 | 10226671 | 10227471 | 1 | MD |
| OTTHUMT00000041703 | RL23A_HUMAN | 6 | 108359697 | 108360151 | 1 | MD |
| OTTHUMT00000042260 | TAAR3_HUMAN | 6 | 132971084 | 132972108 | -1 | RC |
| OTTHUMT00000042682 | N2DL1_HUMAN | 6 | 150362917 | 150367986 | -1 | MS |
| OTTHUMT00000047842 | BMS1_HUMAN | 10 | 46650101 | 46661940 | -1 | MD |
| OTTHUMT00000056207 | SFRS2_HUMAN | X | 34315760 | 34316502 | 1 | MD |
| OTTHUMT00000056993 | Q4G0E3_HUMAN | X | 65211402 | 65212914 | -1 | MS |
| OTTHUMT00000057445 | AP2B1_HUMAN | X | 92363873 | 92366670 | -1 | MD |
| OTTHUMT00000057622 | FOXN3_HUMAN | X | 101688321 | 101689797 | -1 | MS |
| OTTHUMT00000058120 | PA2G4_HUMAN | X | 119886493 | 119887689 | -1 | MC |
| OTTHUMT00000058709 | MAGAB_HUMAN | X | 148538648 | 148539598 | -1 | MD |
| OTTHUMT00000060871 | RL19_HUMAN | X | 149912632 | 149913222 | 1 | MD |
| OTTHUMT00000082555 | TCPZ_HUMAN | 7 | 64162809 | 64171658 | 1 | SD |
| **OTTHUMT00000082689** | ZN630_HUMAN | 7 | 5127408 | 5133903 | -1 | MC |
| OTTHUMT00000099545 | NBPF1_HUMAN | 1 | 147356499 | 147396926 | -1 | MC |
| OTTHUMT00000265424 | PROS_HUMAN | 3 | 90333020 | 90373910 | -1 | MD |
| **OTTHUMT00000269970** | Q14329_HUMAN | 15 | 41018743 | 41019869 | -1 | MC |
| **OTTHUMT00000270027** | MPP10_HUMAN | 15 | 30575493 | 30579834 | -1 | MD |
| OTTHUMT00000280816 | Q5T7C6_HUMAN | 17 | 32574245 | 32575328 | -1 | MR |
| OTTHUMT00000284865 | OR1D5_HUMAN | 17 | 3090720 | 3091658 | 1 | DC |
| *OTTHUMT00000075125* | *Q13731_HUMAN* | *22* | *29931303* | *29931515* | *-1* | *MD* |
| *OTTHUMT00000041268* | *LS14A_HUMAN* | *6* | *76377607* | *76378381* | *-1* | *DC* |
| urn:lsid:pseudogene.org:9606.Pseudogene:17922 | ENSP00000285176 | 3 | 199272756 | 199273767 | -1 | MDC |
| urn:lsid:pseudogene.org:9606.Pseudogene:26822 | ENSP00000345184 | 19 | 59912218 | 59914014 | 1 | MRD |
| urn:lsid:pseudogene.org:9606.Pseudogene:125327 | ENSP00000352019 | 7 | 64164057 | 64170071 | 1 | SD |
| urn:lsid:pseudogene.org:9606.Pseudogene:21057 | ENSP00000265755 | 7 | 65918294 | 65933727 | -1 | SR |
| urn:lsid:pseudogene.org:9606.Pseudogene:22691 | ENSP00000314417 | 10 | 45530590 | 45537239 | -1 | MC |
| urn:lsid:pseudogene.org:9606.Pseudogene:20862 | ENSP00000274599 | 7 | 5127408 | 5133903 | -1 | MC |
| urn:lsid:pseudogene.org:9606.Pseudogene:22401 | ENSP00000341093 | 10 | 31000499 | 31046201 | 1 | MC |
| urn:lsid:pseudogene.org:9606.Pseudogene:143302 | ENSP00000326324 | 17 | 40951004 | 40972397 | -1 | MD |
| urn:lsid:pseudogene.org:9606.Pseudogene:17690 | ENSP00000330021 | 3 | 90337849 | 90366577 | -1 | MD |
| urn:lsid:pseudogene.org:9606.Pseudogene:20201 | ENSP00000296702 | 6 | 90401884 | 90402477 | -1 | MD |
| urn:lsid:pseudogene.org:9606.Pseudogene:25314 | ENSP00000244230 | 15 | 30574414 | 30579839 | -1 | MD |
| urn:lsid:pseudogene.org:9606.Pseudogene:20346 | ENSP00000229708 | 6 | 150362923 | 150364498 | -1 | MS |
| **urn:lsid:pseudogene.org:9606.Pseudogene:18315** | ENSP00000346027 | 4 | 13242542 | 13243320 | -1 | MR |
| urn:lsid:pseudogene.org:9606.Pseudogene:4346 | NUDT9_HUMAN | 10 | 92902340 | 92903337 | -1 | MSRD |
| *urn:lsid:pseudogene.org:9606.Pseudogene:134301* | *ENSP00000370600* | *9* | *130710422* | *130711150* | *-1* | *SRC* |
| urn:lsid:pseudogene.org:9606.Pseudogene:53787 | Q8NA35 | 4 | 166056168 | 166057582 | 1 | MRD |
| urn:lsid:pseudogene.org:9606.Pseudogene:5732 | ADA21_HUMAN | 14 | 69781964 | 69784130 | -1 | MRD |
| urn:lsid:pseudogene.org:9606.Pseudogene:134781 | CXX1_HUMAN | X | 134012862 | 134013487 | -1 | SC |
| urn:lsid:pseudogene.org:9606.Pseudogene:138997 | ENSP00000339841 | 4 | 24382194 | 24383040 | 1 | MR |
| urn:lsid:pseudogene.org:9606.Pseudogene:142888 | ENSP00000339841 | 1 | 116201156 | 116201963 | -1 | MD |
| urn:lsid:pseudogene.org:9606.Pseudogene:20859 | PAPOA_HUMAN | 7 | 4865838 | 4867964 | -1 | MC |
| urn:lsid:pseudogene.org:9606.Pseudogene:2833 | ENSP00000339037 | 6 | 108359706 | 108360148 | 1 | MD |
| urn:lsid:pseudogene.org:9606.Pseudogene:28518 | FOXN3_HUMAN | X | 101688323 | 101689798 | -1 | MS |
| urn:lsid:pseudogene.org:9606.Pseudogene:478 | ENSP00000311430 | 1 | 169918919 | 169920201 | -1 | MS |
| urn:lsid:pseudogene.org:9606.Pseudogene:6132 | HNRPC_HUMAN | 16 | 11244011 | 11244907 | 1 | MC |
| urn:lsid:pseudogene.org:9606.Pseudogene:71776 | ENSP00000268661 | 14 | 56514880 | 56516062 | 1 | MC |
| urn:lsid:pseudogene.org:9606.Pseudogene:7569 | ENSP00000262325 | X | 92363876 | 92366670 | -1 | MD |
| urn:lsid:pseudogene.org:9606.Pseudogene:7695 | CXX1_HUMAN | X | 133983210 | 133983838 | -1 | SC |
| urn:lsid:pseudogene.org:9606.Pseudogene:7371 | ENSP00000333298 | X | 33339054 | 33340208 | 1 | MD |
| urn:lsid:pseudogene.org:9606.Pseudogene:7624 | ENSP00000229239 | X | 108471716 | 108472552 | 1 | MD |
| urn:lsid:pseudogene.org:9606.Pseudogene:2531 | ENSP00000346018 | 6 | 10226671 | 10227468 | 1 | MD |
| ENST00000321205_UB7I3_HUMAN | UB7I3_HUMAN | 8 | 145225524 | 145231128 | -1 | SC |
| URP1_HUMAN--ENST00000358795-432.nt.fasta_548,FERM1_HUMAN | URP1_HUMAN | 20 | 6004669 | 6051677 | -1 | SR |
| NKG2C_HUMAN--ENST00000240618-1.nt.fasta_102 | NKG2C_HUMAN | 12 | 10416857 | 10454012 | -1 | MS |
| FLT3_MOUSE--ENST00000241453-1.nt.fasta_991 | FLT3_MOUSE | 13 | 27475757 | 27572735 | -1 | MS |
| ANKR7_HUMAN--ENST00000357099-1.nt.fasta_173 | ANKR7_HUMAN | 7 | 117652195 | 117669973 | 1 | MS |
| BT3A3_PONPY--ENST00000289361-1568.nt.fasta_177 | BT3A3_PONPY | 6 | 26510465 | 26523414 | 1 | MC |
| APOL3_HUMAN--ENST00000347595-1.nt.fasta_165 | APOL3_HUMAN | 22 | 34866323 | 34962880 | -1 | MD |
| **chr10_Q96RG0.4_-** | Q7KZI7 | 10 | 91587375 | 91589071 | -1 | MDC |
| chr2_P54707.2_+ | P54707 | 2 | 218266718 | 218269632 | 1 | MDC |
| chr8_P39030.1_+ | P61313 | 8 | 130945647 | 130946278 | 1 | MC |

Pseudogene identifiers starting with OTT* are from VEGA; with urn:* are from pseudogene.org; with chr:* are from an earlier study of transcribed processed pseudogenes [Harrison *et al*. 2005]; and the rest are from dmRNA study [Harrison and Yu 2007]. The last column indicates the preservations pattern of the pseudogene in various mammals (‘M’:monkey; ‘D’:dog; ‘S’: mouse; ‘R’: rat; ‘C’: cow). Pseudogene ‘ids’ in bold letters have antisense homology to distant genes, and those in italics are pseudogenes that are transcribed in the antisense direction
